# Optically Stimulated Luminescent Dosimetry for High Dose Rate Brachytherapy

**DOI:** 10.3389/fonc.2012.00091

**Published:** 2012-08-06

**Authors:** Christopher Jason Tien, Robert Ebeling, Jessica R. Hiatt, Bruce Curran, Edward Sternick

**Affiliations:** ^1^Department of Radiation Oncology, Rhode Island HospitalProvidence, RI, USA

**Keywords:** Accuboost, brachytherapy, dosimetry, high dose rate brachytherapy, iridium 192, kilovoltage dosimetry, nanodot, optically stimulated luminescent detectors

## Abstract

**Purpose:** The objective was to determine whether optically stimulated luminescent dosimeters (OSLDs) were appropriate for *in vivo* measurements in high dose rate brachytherapy. In order to make this distinction, three dosimetric characteristics were tested: dose linearity, dose rate dependence, and angular dependence. The Landauer nanoDot™ OSLDs were chosen due to their popularity and their availability commercially. **Methods:** To test the dose linearity, each OSLD was placed at a constant location and the dwell time was varied. Next, in order to test the dose rate dependence, each OSLD was placed at different OLSD-to-source distances and the dwell time was held constant. A curved geometry was created using a circular Accuboost^®^ applicator in order to test angular dependence. **Results:** The OSLD response remained linear for high doses and was independent of dose rate. For doses up to 600 cGy, the linear coefficient of determination was 0.9988 with a response of 725 counts per cGy. The angular dependence was significant only in “edge-on” scenarios. **Conclusion:** OSLDs are conveniently read out using commercially available readers. OSLDs can be re-read and serve as a permanent record for clinical records or be annealed using conventional fluorescent light. Lastly, OSLDs are produced commercially for $5 each. Due to these convenient features, in conjunction with the dosimetric performance, OSLDs should be considered a clinically feasible and attractive tool for *in vivo* HDR brachytherapy measurements.

## Introduction

Optically stimulated luminescence (OSL) is light emission from previously irradiated crystalline materials when stimulated by a light of different wavelength. OSL has been employed for film dosimetry (Schembri and Heijmen, 2007), computed tomography (CT) dosimetry (Yukihara et al., 2009; Ruan et al., 2010), personal dosimetry monitoring (Akselrod et al., 1999; Lee and Lee, 2001; Yukihara and McKeever, 2008), and space dosimetry (McKeever, 2002). In OSL materials, crystal-lattice imperfections are introduced to trap electrons, which are liberated by ionizing radiation (Akselrod et al., 1990; Akselrod et al., 1999; Yukihara and McKeever, 2008).

The materials used for OSL are essentially the same as the more well-known thermoluminescent dosimeters (TLDs), however optically stimulated luminescent dosimeters (OSLDs) are stimulated using light while TLDs are stimulated using heat (McKeever and Moscovitch, 2003). The most common OSL dosimeter material – carbon-doped aluminum oxide (Al_2_O_3_:C) – was originally introduced as a TLD (Akselrod et al., 1990; Yukihara and McKeever, 2008). As an OSLD, aluminum oxide is capable of detecting absorbed dose greater than 10^5^ Gy, with negligible temperature dependence.

There have been varying results regarding angular dependence, which depend on the irradiation environment. Specifically, Jursinic (2010) has measured negligible angular dependence. On the other hand, Kim et al. (2011) measured a response which was 72% higher at an oblique angle than the response when the incident beam was en face to the OSLD. Kerns et al. (2011) measured a milder effect of only 4% lower response in oblique angles vs. en face beam.

Optically stimulated luminescent dosimeters are commonly housed in a plastic container around 100–200 mm^3^. The small size allows placement in cramped spaces which suffer from acute radiation toxicity such as an inframammary fold or between labia. Using physical measurements overcomes the inherent computational challenges of accounting for day-to-day variations, especially in deformable organs such as the breast.

The performance of OSLDs has been well-documented in the megavoltage regime and in the kilovoltage range (Akselrod et al., 1990; Akselrod et al., 1999; Lee and Lee, 2001; McKeever, 2002; McKeever and Moscovitch, 2003; Jursinic, 2007; Schembri and Heijmen, 2007; Yukihara and McKeever, 2008; Reft, 2009; Yukihara et al., 2009; Ruan et al., 2010; Benevides et al., 2011; Kerns et al., 2011; Kim et al., 2011). However, the ability of OSLDs to measure dose in the kilovoltage regime of HDR brachytherapy has not been demonstrated. Furthermore, the OSLD physical sensitive volume must be shown to resolve the high gradients ubiquitous to HDR (Dewerd et al., 2009). This investigation characterized general dosimetric properties of an OSLD in HDR brachytherapy using an Ir-192 source. In addition to quantifying dose linearity and dose rate dependence, a novel method is introduced to measure angular dependence of an OSLD.

## Materials and Methods

**Figure 1:**
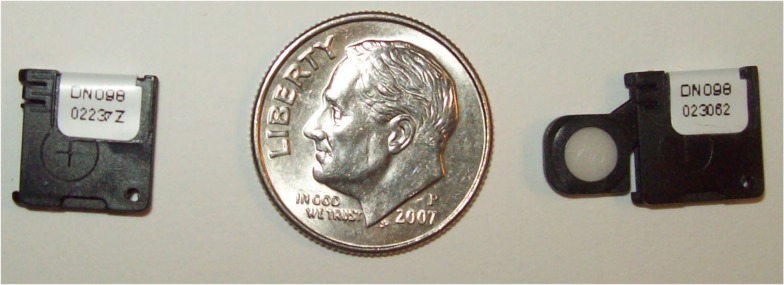
**Two nanoDot™ OSLDs shown to simultaneously display closed (left) and open (right) positions**.

The OSLDs used for this study were a batch of InLight/OSL nanoDot™ (Landauer, Inc., Glenwood, IL, USA) dosimeters. The OSLDs are circular disks with a 5-mm diameter and 0.2 mm thick made of carbon-doped aluminum oxide with a 0.05-mm thick polyester-film cover layer. The disks are housed within a 10 mm × 10 mm× 2 mm light-tight plastic holder. Two OSLDs are shown in Figure 1 below to illustrate the open and closed positions. The manufacturer-specified housing wall-thickness is 0.36 mm and has a density of 1.03 g/cm^3^.

Optically stimulated luminescent dosimeters signals can be read out multiple times. Therefore, for each count measurement, each OSLD was read out 7–10 times in order to reduce statistical noise. A correction factor was applied to account for the read depletion of 0.4% observed by Jursinic (2007). OSLDs were given 16–24 h after irradiation to stabilize (Jursinic, 2007; Reft, 2009; Jursinic, 2010; Kerns et al., 2011; Kim et al., 2011). OSLDs were reused by optically bleaching OSLDs for 24–72 h using a fluorescent lamp (Yukihara et al., 2005; Yukihara and McKeever, 2008; Reft, 2009; Jursinic, 2010).

The brachytherapy source was an Ir-192 seed (half-life: 74 days, average 380 keV emitted energy) with an initial activity around 10,000 mCi (Khan, 2003). Over the course of this investigation, the activity varied between 6,000 and 10,000 mCi as specified by American Association of Physicists in Medicine (AAPM) Task Group (TG) 43 and AAPM TG-56, respectively (Nath et al., 1997; Rivard et al., 2004).

### Dose and dose rate dependence

**Figure 2:**
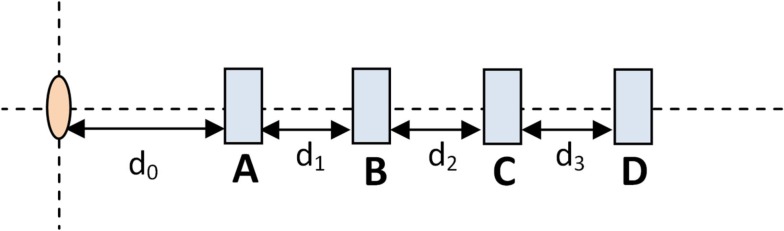
**Dose rate measurement schematic**.

Figure 2 shows the set-up used to test dose linearity and dose rate dependence. The pink oval represents the HDR source while the blue rectangles represent possible OSLD measurement positions. The OSLD’s embossed “Landauer” text was perpendicular to the block and facing toward the catheter.

In order to determine the dose dependence, an OSLD was placed in location B and the dwell time was varied. The distance between source and OSLD remained constant to maintain constant dose rate. On the other hand, in order to test the dose rate dependence, the dwell time was held constant and the OSLD was placed in one of the four different locations. By the inverse-square law, different OSLD-to-source produced varying dose rates.

By placing the device perpendicular to the scan axis, a CT scan of the dose rate measurement device was used to give sub-millimeter resolution measurements of exact OSLD locations. The OSLD locations are 1.88, 2.85, 3.88, 4.92, 5.87, 6.88, 7.86, 8.89, and 9.83 cm from the source. The CT dataset was imported into the HDR treatment planning software, Oncentra version 4.0 (Nucletron B.V., Veenendaal, Netherlands).

The exact position of the HDR Ir-192 source within the catheter itself was localized using radiochromic (GAFCHROMIC, Ashland, Covington, KY, USA) film. This resulted in a 2 mm shift, which is reasonable given the Ir-192 source schematics described by Daskalov et al. (1998).

### Angular dependence

**Figure 3:**
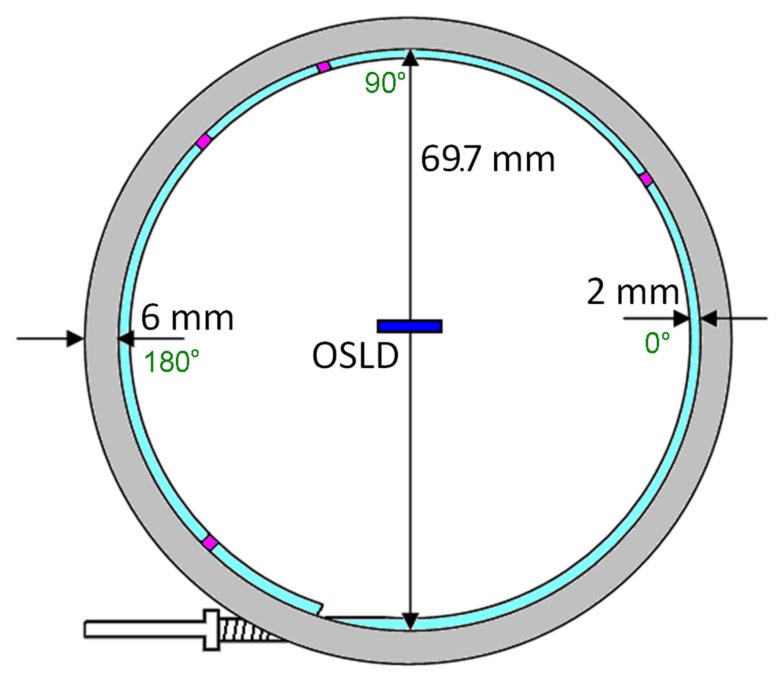
**Angular dependence test using circular Accuboost^®^ applicator**.

A circular AccuBoost^®^ (Advanced Radiation Therapy, LLC., Tyngsboro, MA, USA) applicator was chosen to provide a rigid circular shape for angular dependence measurements. AccuBoost^®^ procedures use an HDR Ir-192 source in this applicator to deliver radiation to breast lesions (Rivard et al., 2009). To minimize the anisotropy of an HDR source, the applicator positions the source’s long axis normal to the applicator’s central axis (Nath et al., 1995, 1997). The applicator has been shown to be large enough for a high dose rate, yet small enough to approximate as a point source. Figure 3 shows the applicator’s dimensions alongside the OSLD’s (dark blue rectangle) – and four dwell locations (purple blocks).

The applicator diameter was measured to be 69.7 mm. A Monte Carlo study by Rivard et al. (2009) modeled the AccuBoost^®^ source as a cylinder 0.65 mm in diameter and 3.6 mm long. The 70 mm applicator is approximately one order larger than the source (3.6 mm); therefore, the seed was approximated as a point source. This assertion will be discussed later.

## Results

### Dose linearity

**Figure 4:**
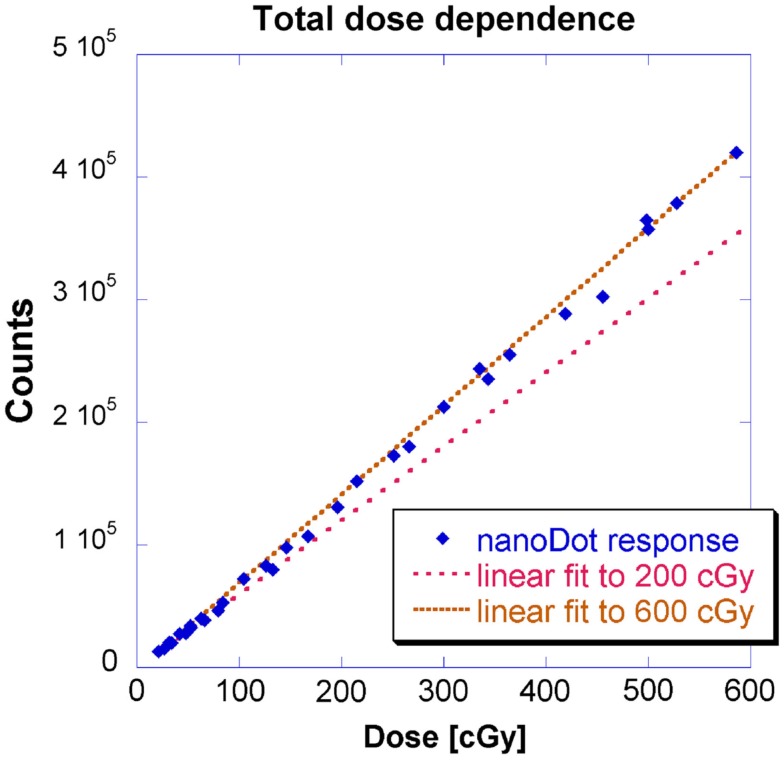
**Counts vs. dose, using a constant dose rate, for a larger total dose**.

This geometry corresponds to Figure 2 where measurements were made at location B and the source-to-detector distance remained constant. Figure 4 shows doses from 0 to approximately 200 cGy where the relationship between counts and dose has been previously shown to be linear (Kerns et al., 2011; Kim et al., 2011). This investigation yielded similar results: a linear regression modeled the relationship up to 200 cGy with a coefficient of determination (*R*^2^) of 0.9997 and a slope of 600 counts/cGy. The coefficient of variation of the individual OSLD measurements was below 0.02 for each OSLD used.

Previous studies have simply stated that OSLDs possess a supralinear relationship between dose and counts above 200 cGy (Kerns et al., 2011; Kim et al., 2011). However, HDR brachytherapy entails a larger doses up to around 500 cGy. Therefore, this investigation measured a second region up to 600 cGy. Figure 4 shows the results for a linear fit to measurements up to 600 cGy alongside the 200 cGy linear fit presented earlier in Figure 4. A linear regression fit to 600 cGy had a slightly lower *R*^2^ of 0.9988 and a larger slope of 725 counts/cGy.

The lower limit of dose linearity was not investigated because HDR delivers such large doses expected in most HDR brachytherapy applications (Nath et al., 1997). However, the lowest dose measured at 20 cGy remained in the linear fit for both 200 and 600 cGy thresholds; also, extrapolating to background count levels (200 counts) corresponded to 0.3 cGy. Regardless, low dose levels are problematic to deliver because of the short dwell times: a more rigorous timer accuracy would be required than the 2% guideline established by Nath et al. (1997). Secondly, there is significant relative contribution due to the finite transit time.

The authors note that there may be better models for the OSLD response. However, the linear model fit with an *R*^2^ larger than 0.99. Clinically, a linear model was preferred because it requires only a single fitted parameter.

### Dose rate dependence

**Figure 5:**
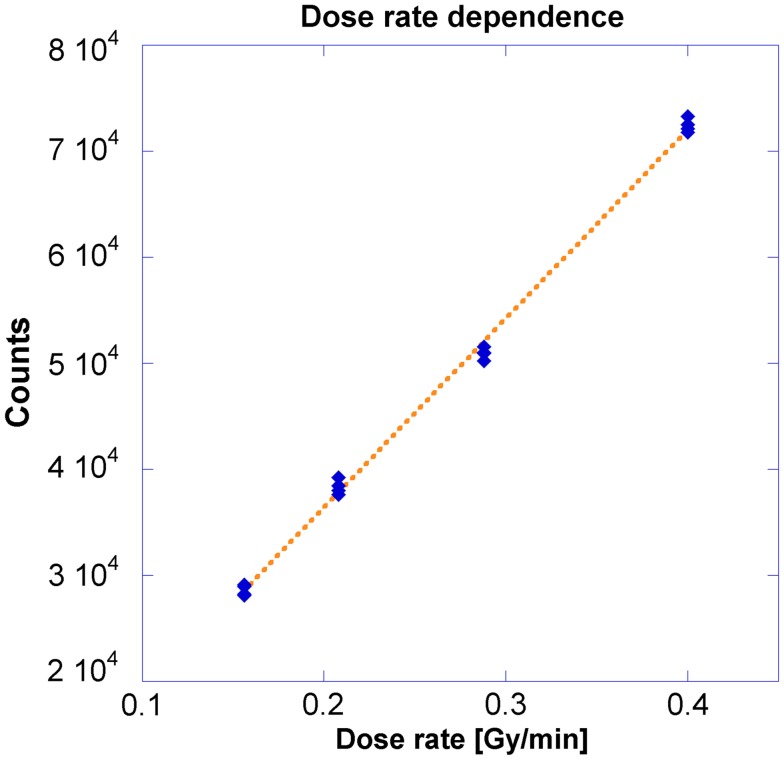
**Relationship between counts and dose, using different dose rates**.

This geometry corresponds to Figure 2 where measurements were made at locations A through D. Different dose rates were obtained as dictated by the inverse-square law. A 250 s dwell time was required to deliver 100 cGy to the OSLD placed at the nominal 5 cm position. Figure 5 demonstrates the OSLD’s independence to dose rate by showing the relationship between OSLD response and the dose rate, with overall *R*^2^ of 0.999. Measurements agreed well with the theoretical dose as calculated using TG-43 and inverse-square factor calculated using the positions summarized previously in Section II.A, with a maximum error of 3.9% relative to the measurements (Nath et al., 1995). The computational output factors calculated by Oncentra agreed well, with a maximum error of 2.3% relative to the measurements.

### Angular dependence

Measurements by the Nucletron Source Position Simulator tool showed the source traveled 211.0 mm. Using the previously measured diameter of 69.7 mm and the catheter radius of 1 mm in, the source would travel 212.6 mm for a full rotation. However, the end of the catheter was sealed and the applicator needed to have some sort of entry point – thereby traversing only 356° out of 360°. Instead of 212.6 mm, the source will travel 210.2 mm from the beginning to end of the applicator. The ratio between angle and the source travel distance was determined to be 1.7°/mm. The position-to-angle ratio was tested by using a laser to examine the coincidence of the 0°and 180° dwell positions within the applicator. Future simulations should model the source as a torus with a 67.7 mm major diameter and 0.3 mm minor diameter over only 356° out of 360°.

**Figure 6:**
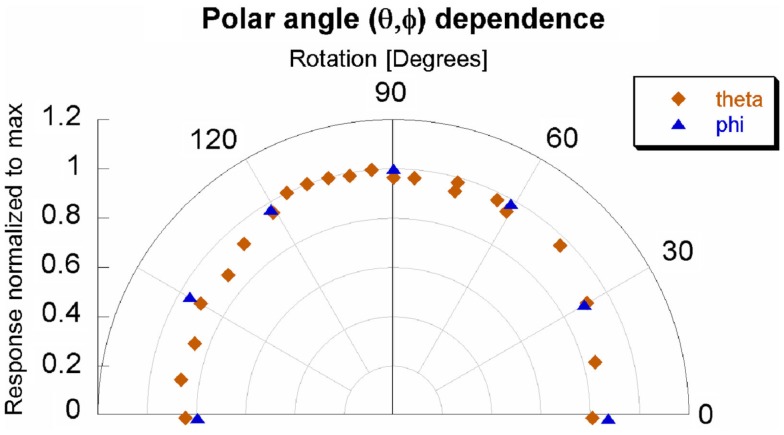
**Angular dependence of OSLD**.

**Figure 7:**
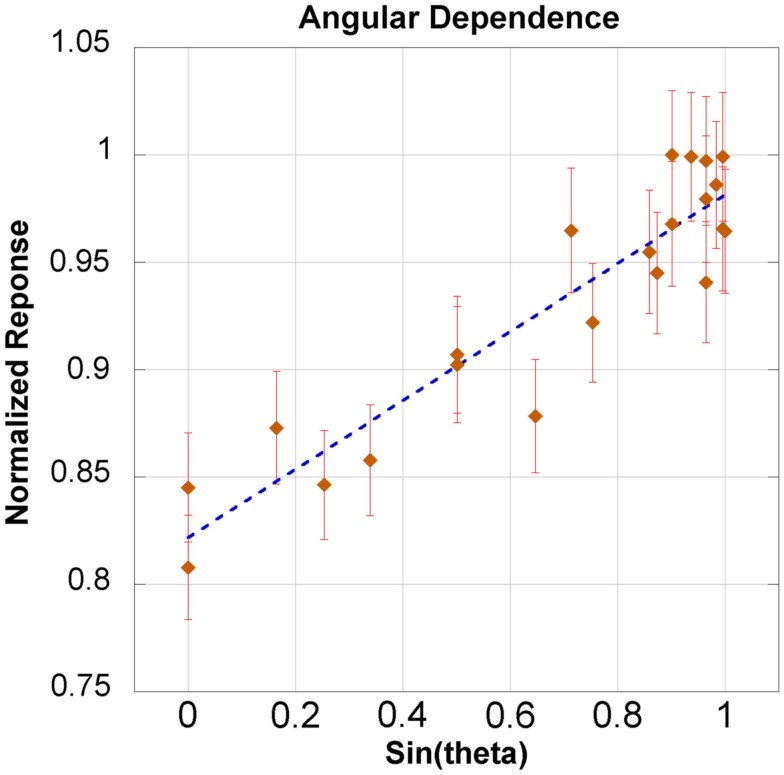
**Angular dependence fitted to sin(θ)**.

The dependence for both azimuthal and transverse angular planes is shown in Figure 6. There was a slight angular dependence most noticeable near 0° and 180° – representing an “edge-on” irradiation – where response dropped up to 20%. However, at an incident angle of 30° the drop in response is only 10%. The slight asymmetry of Figure 6 will be addressed in the discussion section. The response taken as a function of the sine of the angle yielded an acceptable linear fit, with an *R*^2^ of 0.858. This model is shown in Figure 7 for the theta plane.

## Discussion

Using physical measurements avoids potential pitfalls found in Monte Carlo modeling of HDR such as source construction, movable components, source emission, and source activity distribution (Dewerd et al., 2009). Physical measurement accuracy was most dependent upon volume averaging, self-attenuation, and absorbed dose sensitivity (Dewerd et al., 2009). In particular, OSLDs can avoid volume averaging issues by placing the face orthogonal to the anticipated gradient, thus giving resolution of 0.2 mm – the width of the disk.

### Dose dependence

This investigation also observed linearity in the kilovoltage HDR energy regime with for doses up to 600 cGy. The observed linear-supralinear behavior between the 200 and 600 cGy thresholds is attributed to a residual radiation-induced signal due to the filling of deeper traps in the detector (Jursinic, 2010). The focus of this investigation was not to establish an absolute calibration factor *per se*, but to investigate the fit of a linear model for OSLDs in the kilovoltage energy range. In principle, with two measurements, a linear model could be extrapolated to completely characterize response up to 600 cGy.

### Dose rate dependence

Previous OSLD studies have been conducted on linear accelerators where dose rate is determined by the pulse rate. In other words, the dose rate per unit second is varied, but the “instantaneous dose” – dose per pulse – does not change (Khan, 2003). In this investigation, the instantaneous dose rate was changed through variation of the source-to-detector distance and the relationship between counts and dose rate was found to be linear. Table 1 shows the dose calculated through either TG-43 calculations or Oncentra calculations is consistent with measured results, with a maximum discrepancy of 3.9%, which is still below the clinically acceptable threshold of 5% (Nath et al., 1995; Khan, 2003; Klein et al., 2009).

**Table 1:** **Dose rate comparison between measurement vs. theoretical calculations and Oncentra calculations**.

Nominal position	Avg counts measured	Avg counts normalized	Inverse-square	% Diff (Inv sq)	Oncentra calculation	% Diff (Oncentra)
5	72388.3	1.000	1.000	–	1.000	–
6	50908.5	0.703	0.703	0.0	0.710	0.9
7	38428.3	0.531	0.511	3.9	0.519	2.3
8	28443.3	0.393	0.392	0.2	0.389	1.0

### Angular dependence

The angular dependence of the system appears to be asymmetric. The most probable, explanation for the asymmetry is the positioning of the OSL material within its casing. Specifically, the OSLD was positioned at the center of the applicator. As can be inferred from Figure 1, the center of the sensitive disk is actually not coincident with the center of casing – in fact, the two centers were approximately 1.5 mm apart in both lateral dimensions. The OSL material is located behind inscribed cross-hairs which can be seen in the left side of Figure 1, immediately below the sticker. In order to better reflect a clinical set-up, the OSLD was positioned relative to the plastic casing rather than the sensitive OSL material. Again, this set-up was purposely chosen to represent the scenario which would be implemented for clinical practice. Ideally, the angular dependence would not be relevant because the irradiation would be immediately en face to the OSLD.

The next reason for the asymmetric angular dependence would be the approximation of the Ir-192 seed as a point source. For the angular dependence tests within the Accuboost applicator, the OSLD-to-source distance was 35 mm. The actual OSLD material is a 2-mm disk. Therefore, the OSLD-to-source distance was approximately one order of magnitude larger than the detector. Strictly speaking, the Ir-192 seed is small line source with the OSLD off-center and closer to one side of the Accuboost applicator. Comparing the effective pathlengths using a point source approximation and a perfectly centered OSLD leads to a maximum uncertainty of 4.4%, with a mean uncertainty of approximately 1.8%.

The last reason for the asymmetric angular dependence would be the elastic stiffness of the HDR source: by definition, any cable which traverses 90° will be tangential to a wall at least once. This contact is dictated by the radius of curvature. This could lead to the source not positioned as expected in terms of both orientation and location, which would lead to an uncertainty of approximately 0.5 mm in a radial direction.

Figure 7 shows the linear relationship between the response and the sine of the θ angle. The coefficient of determination was 0.858. Additionally, the linear model was within the error bars for 20 out of 21 measurements. The maximum loss of response was observed in only an “edge-on” scenario. This investigation recommends maintaining an incident angle greater than 30° – where there is only a 10% drop in response. An angular correction factor could theoretically be determined from Figure 7, especially if an edge-on scenario was clinically unavoidable.

### Clinical relevance and anticipated limits

#### TLDs vs. OSLDs

Both TLD and OSLD measurements are based upon materials measure the amount of visible light emitted from the material after exposure to ionizing radiation. While TLDs have been historically used for clinical *in vivo* dosimetry measurements for many years, many clinics have replaced TLDs with OSLDs (Low et al., 2011). In fact, OSLDs have become so predominant that the Radiological Physics Center (RPC), the governing institution for credentialing clinical radiotherapy units changed in 2010 from TLDs, in use since 1968, to OSLDs for annual output checks (Low et al., 2011). OSLD measurements are available in less time and faster than with TLDs (Yukihara et al., 2005; Mayles et al., 2007). McKeever and Moscovitch (2003) have written a comprehensive article which examines OSLDs in comparison with TLDs.

In order to be properly read, TLDs require about 24 h for stabilization and can only be read once (Akselrod et al., 1990). Additionally, due to their delicate construction, TLDs are difficult to handle and the process of read out is extremely time consuming (Yukihara and McKeever, 2008). For example, TLDs require vacuum tweezers, scales, nitrogen, and annealing ovens (Yukihara and McKeever, 2008). Additionally, the clinic must employ control TLDs and glow curves which are unique to each set of measurements (Akselrod et al., 1990).

Optically stimulated luminescent dosimeters are read out using light, which is much faster, more precise, and, in general, more reproducible than heating, as used by TLDs (Yukihara et al., 2005). Additionally, OSLD dose curves are established by the vendor prior to shipping which precludes establishing a control set of OSLDs for every batch (McKeever, 2002). Accounting for read depletion, OSLDs can be re-read and there are currently clinics which store OSLDs used to establish a permanent record (McKeever, 2002).

#### OSLD clinical advantages

As previously discussed, OSLDs provide significant time-savings for the medical physicist, however these detectors also provide a superb detector choice for physicians. For example, due to their thin disk size, OSLDs can be placed in inframammary fold or between labia, which are common sites of significant acute radiation toxicity. These regions frequently possess high dose gradients and with careful placement, OSLDs can be used without noticeable volume averaging effects. Specifically, OSLDs should be placed with its face normal to the direction of the dose gradient. And, finally, angular dependence appears to be noteworthy only in “edge-on” scenarios, which can be avoided in most clinical situations.

HDR brachytherapy is used at this particular institution for a wide range of treatment techniques including tandem and ovoid treatments for cervical cancer, balloon brachytherapy for accelerated partial breast irradiation (APBI), intrabronchial insertions for constrictive upper airway disease, Freiburg flap applications for superficial cancers, and wide range of interstitial applications. This institution prefers to use Accuboost^®^ for the boost doses in breast cases to overcome issues of deformable breast volume (Oh et al., 2006; Yang and Rivard, 2009). Hence, the circular Accuboost^®^ applicators were available.

While calculated dose to the targeted tissue is reliable, the dose to other organs such as skin is not trivial. Dose to the skin surface is of significant consequence as acute toxicity may lead to treatment breaks and if severe may also lead to consequential late effects such as telangectasias and subcutaneous fibrosis, as sometimes found in APBI (Yang and Rivard, 2010). The large majority of HDR brachytherapy doses are prescribed around 300–500 cGy per fraction. APBI prescription and skin dose are 340 and 495 cGy, respectively (Yang and Rivard, 2010). In the future, if anticipated doses delivered will be higher, measurements can determine whether the behavior is linear past 600 cGy. As seen in dose rate measurements, these OSLDs showed the ability to handle the high dose rates even at 5 cm, in air. In a clinical case, there would also be soft tissue attenuation reducing the dose rate.

## Conclusion

This work is the first investigation of the use of OSLDs explicitly using HDR sources: testing dose linearity, dose rate capabilities, and angular response. The OSLDs have been shown to overcome unique challenges of HDR brachytherapy, namely high dose gradients and high dose rate. With regards to the high dose gradients, due to its physical dimensions, the OSLDs is able to resolve gradients as thin as 0.2 mm. Also, due to their small size, OSLDs avoids the pitfalls of many HDR brachytherapy detectors such as volume averaging and limited spatial resolution.

This investigation did not focus on measuring an absolute calibration factor, but instead established the linearity of OSLDs up to 600 cGy. In other words, for the majority of clinical applications, linear interpolation is appropriate. The OSLDs have been shown to perform with a linear response at high dose rates in air. There was an angular dependence observed which became significant in “edge-on” scenarios where the radiation source was directly incident upon the edge of the OSLD. This was attributed to the purposeful shift in order to align the center relative to the OSLD casing rather than the OSL sensitive material. Regardless, it is recommended that the angle of incidence remains larger than 30°, but it is possible to derive an angular correction factor if oblique angles are unavoidable using a linear model between response and the sine of the incident angle.

Patient positioning can vary significantly day-to-day, making accurate computational modeling of all tissues at risk quite difficult, especially those not immediately adjacent to the treated tissue. Using physical measurements is a practical solution because OSLDs are small and provide an almost “point” detector geometry. While TLDs have been historically used for many years, recently many clinics have recognized the convenience and reliability of OSLDs and replaced TLDs. Specifically, the RPC, the governing institution for credentialing clinical radiotherapy units changed in 2010 from TLDs, in use since 1968, to OSLDs for annual output checks.

Optically stimulated luminescent dosimeters have an advantage in their convenient and time-saving read out; afterward, they can be re-read, serve as a permanent record, or reused by annealing with conventional fluorescent light. Additionally, OSLDs are commercially available – in bulk if necessary – for around $5 each. For these reasons, combined with their dosimetric performance shown in this investigation, OSLDs should be considered a feasible and attractive tool for *in vivo* HDR brachytherapy measurements.

## Conflict of Interest Statement

The authors declare that the research was conducted in the absence of any commercial or financial relationships that could be construed as a potential conflict of interest.
